# The Receptor-Binding Domain in the VP1u Region of Parvovirus B19

**DOI:** 10.3390/v8030061

**Published:** 2016-02-24

**Authors:** Remo Leisi, Chiarina Di Tommaso, Christoph Kempf, Carlos Ros

**Affiliations:** 1 Department of Chemistry and Biochemistry, University of Bern, Bern 3012, Switzerland; chiarina.ditommaso@hotmail.com (C.D.T.); christoph.kempf@dcb.unibe.ch (C.K.); carlos.ros@dcb.unibe.ch (C.R.); 2 CSL Behring AG, Bern 3014, Switzerland

**Keywords:** parvovirus B19, virus entry, VP1u, receptor-binding domain, immunodominant region

## Abstract

Parvovirus B19 (B19V) is known as the human pathogen causing the mild childhood disease *erythema infectiosum*. B19V shows an extraordinary narrow tissue tropism for erythroid progenitor cells in the bone marrow, which is determined by a highly restricted uptake. We have previously shown that the specific internalization is mediated by the interaction of the viral protein 1 unique region (VP1u) with a yet unknown cellular receptor. To locate the receptor-binding domain (RBD) within the VP1u, we analyzed the effect of truncations and mutations on the internalization capacity of the recombinant protein into UT7/Epo cells. Here we report that the N-terminal amino acids 5–80 of the VP1u are necessary and sufficient for cellular binding and internalization; thus, this N-terminal region represents the RBD required for B19V uptake. Using site-directed mutagenesis, we further identified a cluster of important amino acids playing a critical role in VP1u internalization. *In silico* predictions and experimental results suggest that the RBD is structured as a rigid fold of three α-helices. Finally, we found that dimerization of the VP1u leads to a considerably enhanced cellular binding and internalization. Taken together, we identified the RBD that mediates B19V uptake and mapped functional and structural motifs within this sequence. The findings reveal insights into the uptake process of B19V, which contribute to understand the pathogenesis of the infection and the neutralization of the virus by the immune system.

## 1. Introduction

Parvovirus B19 (B19V) is a small, non-enveloped virus belonging to the genus Erythroparvovirus [1,2]. Infection in children leads to the characteristic childhood disease *erythema infectiosum*, also known as fifth disease [3,4,5]. While B19V infection in healthy individuals often has a mild or asymptomatic course, the infection in immunocompromised persons, pregnant women, or in patients with hemolytic disorders may lead to severe manifestations [6]. Furthermore, B19V infection can be associated with disorders as rheumatoid arthritis [7,8], hepatitis [9], vasculitis [10], and inflammatory cardiomyopathies [11,12]. Productive viral replication takes place in the bone marrow, where cells from the intermediate erythroid differentiation stages CFU-E to early erythroblasts represent the main target [13,14,15]. The extraordinary tropism of B19V is mainly determined by a selective uptake into these erythroid progenitors and early precursors [16]. The glycosphingolipid globoside (Gb4Cer) has been identified as a cellular receptor for B19V [17], serving as an attachment factor for the virus on the cell surface [18]. However, the broad expression pattern of Gb4Cer and the proposed co-receptors α5β1 integrin [19] or Ku80 [20] cannot explain the extremely restricted viral uptake. This suggests a still unknown receptor responsible for the specific viral internalization into early erythroid precursors, which defines the narrow tropism of B19V.

In addition to the highly selective virus uptake based on capsid-receptor interactions, previous studies have shown that an antibody-dependent uptake of B19V into other tissues may occur [12,21]. This antibody-dependent binding/uptake mechanism may explain the detection of B19V in endothelial or immune cells and can further explain the inflammatory immune responses in these tissues, leading to the typically associated diseases of arthritis, vasculitis, and myocarditis [12,22]. Importantly, the antibody-dependent entry into these cells occurs after the initial infection and does not account for a productive viral replication.

The capsid of B19V is an icosahedral assembly of 60 protomers, of which 95% are viral protein 2 (VP2) and 5% viral protein 1 (VP1) [23]. VP1 consists of the entire VP2 sequence plus an additional N-terminal region of 227 amino acids (AA): the VP1 unique region (VP1u). Despite its minor number in the capsid, the VP1u represents an immunodominant part of the virus. Upon B19V infection, the primary humoral immune response predominantly raises antibodies against the major capsid protein VP2, neutralizing the virus by targeting conformational epitopes on the capsid surface. Long-term immunity, however, selects an increasing proportion of antibodies against epitopes in the VP1u region [24,25,26,27]. Studies with immunized rabbits have shown that the most neutralizing antisera are produced with peptides from the N-terminal part of the VP1u [28,29]. The clustering of neutralizing epitopes in the VP1u from AA 20-80 suggests the presence of important functional motifs in this region. Consistent with this hypothesis, we recently found that the VP1u region is responsible for the restricted B19V entry into susceptible cells [16]. Furthermore, we used the VP1u to design a viral-based delivery system, which specifically targets cargo molecules as fluorescent dyes, DNA and toxic payloads to intermediate erythroid differentiation stages and erythroleukemic cells [30]. This finding suggests that the VP1u of B19V binds a still unknown receptor, whose expression is highly restricted to erythroid precursors around the proerythroblast differentiation stage (CFU-E to early basophilic erythroblast). Previous results have shown that the N-terminal 100 amino acids of the VP1u are sufficient for the specific binding and internalization into these cells [16]. However, the critical amino acids involved in the receptor binding and the molecular aspects of the interaction were not elucidated.

In this work, we sought to define the RBD of the VP1u, and to map functional and structural motifs within this sequence. To address these questions, we predicted *in silico* the secondary structure and conserved amino acid clusters within the VP1u. Based on this prediction, we truncated and mutated the recombinant protein and tested its binding and internalization capacity in UT7/Epo cells. The results provide novel insights into the internalization process of B19V and further explain the efficient neutralization of B19V by antibodies against the VP1u region.

## 2. Materials and Methods

### 2.1. Cells and Viruses

UT7/Epo cells were kindly provided by Eiji Morita (Tohoku University School of Medicine, Sendai, Japan) and grown in RPMI 1640, 5% fetal calf serum (FCS), 2 U/mL recombinant human erythropoietin (rhEPO), supplemented with l-glutamine and penicillin/streptomycin. Human plasma from a parvovirus B19 infected person (5 × 10^9^ virions/µL, genotype 1) was obtained from our donation center (CSL Behring AG, Charlotte, NC, USA).

### 2.2. Mutation and Expression of Recombinant VP1u

The VP1u sequence originally derived from the infectious clone pB19-M20 (Susan Wong, National Institutes of Health, Bethesda, MD, USA) and was cloned into the pT7-FLAG-MAT-Tag-2 expression vector (Sigma, St. Louis, MO, USA) as previously described [16]. Mutations and truncations were introduced by QuikChange PCR (Agilent Technologies, Santa Clara, CA, USA) and confirmed by sequencing. The expression of the recombinant VP1u protein was carried out in BL21(DE3) *E. coli* and lysogeny broth media containing ampicillin. The bacteria culture was grown until an OD_600_ of ~0.5 to induce then protein expression with 1 mM isopropyl-β-d-thiogalactopyranoside (IPTG) for 4 h at 37 °C. The pelleted cells were resuspended in phosphate buffered saline (PBS, pH 8) with 5 mM imidazole, and sonicated for 15 × 10 s. Cell debris was removed by centrifugation at 4 °C, 12000× *g*. The recombinant VP1u in the soluble fraction was purified under native conditions with nickel nitrilotriacetic acid (Ni-NTA) agarose and eluted with 250 mM imidazole in PBS. To obtain high purity of the VP1u, we diluted the eluate (1/30 in PBS) and purified again with Ni-NTA agarose. The final quantity and purity of the recombinant VP1u were analyzed by NanoDrop and SDS PAGE. The expression and purification of one liter bacteria culture yielded ~10 mg pure VP1u.

### 2.3. Binding and Internalization of the Recombinant VP1u in UT7/Epo Cells

UT7/Epo cells (5 × 10^5^) were harvested and incubated with recombinant VP1u-FLAG (200 ng; ~10^13^ molecules) at 4 °C. For following immunofluorescence detection, 0.25 µg of the rat αFLAG antibody (Agilent Technologies) was added to the sample to label the VP1u. To inhibit binding of the primary αFLAG antibody to Fc receptors and thus to avoid VP1u-independent signal, we pre-blocked the cells for 10 min at 4 °C with 0.4 µg of a mouse αCD32 (FcγRII) antibody (BD Biosciences, San Jose, CA, USA). For binding assays, UT7/Epo cells were kept at 4 °C for 1 h and then washed four times with ice-cold PBS. For internalization, the cells were incubated at 37 °C for 1 h and washed twice. To remove non-internal VP1u, we trypsinized the cells for 4 min at 37 °C and further washed the pelleted cells twice with PBS. The cells were either fixed for immunofluorescence or resuspended in Laemmli buffer for Western blot detection. Immunofluorescence detection of the VP1u, labeled already with αFLAG antibody (rat), was carried out with secondary goat anti-rat antibody-Alexa Fluor 488 (Agilent Technologies) and visualized by confocal microscopy (Laser Scanning Microscope LSM 510; 63× magnification objective, Carl Zeiss, Jena, Germany). Detection of VP1u in Western blots was performed with αFLAG antibody (rat) and secondary HRP-labeled anti-rat antibody (goat).

### 2.4. Competition of B19V with Recombinant VP1u

UT7/Epo cells (5 × 10^5^) were incubated with 10^11^ B19 virions in the presence or absence of recombinant VP1u protein. To achieve significant competition, we applied a 200-fold molecular excess of free VP1u (400 ng of ∆C126, ~2 × 10^13^) *vs.* B19V. Viral internalization was allowed for 40 min at 37 °C. After removal of non-internalized virions by washes and trypsinization, the internalized viral DNA was extracted (DNeasy Blood and Tissue Kit; Qiagen, Hilden, Germany) and quantified by iTaq SybrGreen qPCR (BioRad, Hercules, CA, USA; forward primer: 5′-GGGCAGCCATTTTAAGTGTTT-3′; reverse primer: 5′-CCAGGAAAAAGCAGCCCAG-3′).

### 2.5. VP1u-Fluorescein Conjugation

Recombinant ∆C126 VP1u (1 mg/mL) was labeled with a 20-fold molecular excess maleimide-fluorescein (Thermo Scientific, Waltham, MA, USA) in PBS (pH 7) and 5 mM tris(2-carboxyethyl)phosphine (TCEP). The coupling reaction was performed for 2 h at room temperature and overnight at 4 °C. The VP1u-fluorescein conjugate was purified with Ni-NTA and the crosslinking was verified by SDS PAGE.

### 2.6. VP1u-DNA Conjugation with Sense and Antisense Oligonucleotides

The complementary oligonucleotides for VP1u-conjugation were designed with a minimal length to allow specific qPCR (39 nt), and with a sequence that does not allow self-hybridization. For Click Chemistry conjugation with the VP1u, the sense and antisense oligonucleotides (39 nt) were modified with an azide residue either at the 5′ or 3′ of the oligonucleotide (azide-5′-GACTGGGACGCTGGACTGACCGGAGAGGTGGTGGAGGAG-3′ (sense); 5′-CTCCTCC ACCACCTCTCCGGTCAGTCCAGCGTCCCAGTC-3′-azide (antisense)). Oligonucleotide synthesis and azide-modification were provided by Microsynth. The azide-modified oligonucleotides (0.2 mM) were coupled to 10 mM alkyne-PEG_4_-maleimide using Cu(I) catalysis (0.5 mM CuSO_4_, 0.5 mM tris(benzyltriazolylmethyl)amine (TBTA), 0.5 mM ascorbate)). The reaction was performed in 50% DMSO, 150 mM phosphate buffer (pH 6.75) for 3 h at room temperature. The maleimide-activated oligonucleotides were pelleted by acetone precipitation (85% acetone, −20 °C) and washed with pure acetone. The purified, washed and dried pellet of maleimide-activated oligonucleotides was resuspended in 150 mM phosphate buffer (pH 6.75, 0.2 mM final concentration of oligonucleotides) and incubated with reduced ∆C126 (0.01 mM) to achieve VP1u-DNA conjugation (overnight at 4 °C, 5 mM TCEP). VP1u-DNA conjugates were further purified by Ni-NTA agarose and analyzed for coupling efficiency by SDS PAGE and Western blot. Binding and internalization experiments with VP1u-DNA conjugates were performed as specified above. Since DNA extraction with silica columns does not allow isolation of short oligonucleotides, the internalized VP1u-DNA (39 nt) was prepared for qPCR by cell lysis (Triton X-100) and Chelex^®^ treatment. Quantitative PCR of the VP1u-DNA (39 nt) was performed with the primers 5′-GACTGGGACGCTGGAC-3′ (forward) and 5′-CTCCTCCACCACCTCTC-3′ (reverse).

### 2.7. Statistics from Quantitative Methods

Values show means ± standard deviations. Replicates represent values from independent experiments.

### 2.8. In Silico Experiments

Amino acid alignments of the erythroparvovirus family members were carried out with ClustalW2. The secondary structure of the ∆C126 VP1u was predicted using the JPred server [31]. *Ab initio* tertiary structure prediction of the RBD was performed using the QUARK server [32].

## 3. Results

### 3.1. The Receptor-Binding Domain (RBD) of the VP1u Spans the N-Terminal Amino Acids 5–80

**Figure 1:**
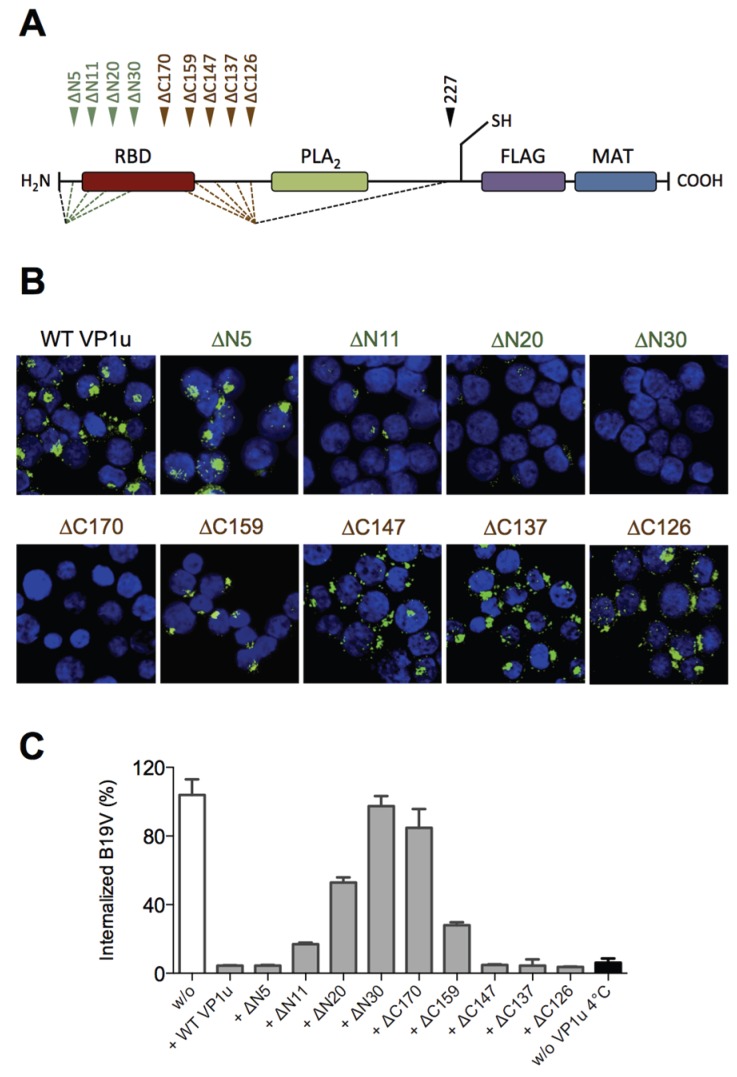
Localization of the receptor-binding domain (RBD) within the VP1u. (**A**) Schematic depiction of the recombinant VP1u protein (227 AA) and the introduced truncations. N-terminal truncations (green): ∆N5, ∆N11, ∆N20, ∆N30. C-terminal truncations (brown): ∆C126, ∆C137, ∆C147, ∆C159, ∆C170. Motifs: Receptor-binding domain (RBD, brown); phospholipase A2 (PLA2, AA 130–195, green [33]); introduced unique cysteine (C228); FLAG tag for immunodetection (violet); metal affinity tag (MAT) for Ni-NTA-purification (blue); (**B**) Representative immunofluorescence images of VP1u internalization (green signal) into UT7/Epo cells. VP1u protein variants were internalized at 37 °C for 1 h and detected by αFLAG antibody; (**C**) Quantification of B19V uptake in presence of VP1u protein variants as competitors (*n* ≥ 2). Values were normalized to unhindered internalization without VP1u (white bar). Control for no internalization (black bar) was kept at 4 °C instead of incubation at 37 °C. All samples shown in (B,C) were trypsinized after incubation at 37 °C to remove uninternalized VP1u in (B) and B19V in (C), respectively.

Our previous results showed that the N-terminal 100 AA of the VP1u (∆C126) are sufficient for the specific binding and internalization into B19V susceptible cells [16]. To more precisely define the RBD of the recombinant VP1u, we introduced serial truncations of about 10 AA at the N- and C-terminus (Figure 1A; Table 1), and expressed the truncated VP1u as previously described [16]. The uptake capacity of the truncated VP1u proteins into UT7/Epo cells was analyzed by immunofluorescence and in competition assays with B19V. Internalization of the VP1u was performed by incubation of UT7/Epo cells with the recombinant VP1u for 1 hour at 37 °C. The cells were washed, trypsinized and fixed for immunostaining. The FLAG-tagged VP1u was stained with αFLAG antibody and AlexaFluor 488 labeled secondary antibody. The fluorescent signal of intern-alized VP1u was visualized by confocal microscopy (Figure 1B). The results show that the VP1u variants internalized when they were truncated less than 5 AA at the N-terminus or less than 147 AA at the C-terminus; longer truncations at both ends resulted in impaired VP1u internalization. 

Competition of B19V uptake by recombinant VP1u protein was performed on UT7/Epo cells as previously described [16]. To achieve effective competition of B19V uptake, the recombinant VP1u was added as a 200-fold molecular excess compared to B19 virion concentration (one virion contains ~3 VP1u per capsid). Cellular uptake of B19V in presence of VP1u proteins was allowed for 1 h at 37 °C. After internalization, cells were washed, trypsinized and viral DNA was extracted for quantitative PCR. The results show that the functional recombinant VP1u proteins efficiently blocked B19V uptake. However, longer than 5 AA truncations at the N-terminus or 147 AA at the C-terminus of the VP1u resulted in a gradual decrease of the competitive capacity (Figure 1C; Table 1), consistently fitting with the observed internalization capacity in Figure 1B. 

Taken together, the results of VP1u internalization and B19V uptake competition demonstrate that the VP1u region between AA 5–80 represents the RBD (Figure 1A), which mediates the uptake into UT7/Epo cells.

### 3.2. The Conserved Amphiphilic Helix 1 (AA 14–31) Plays a Crucial Role for the Internalization

**Figure 2:**
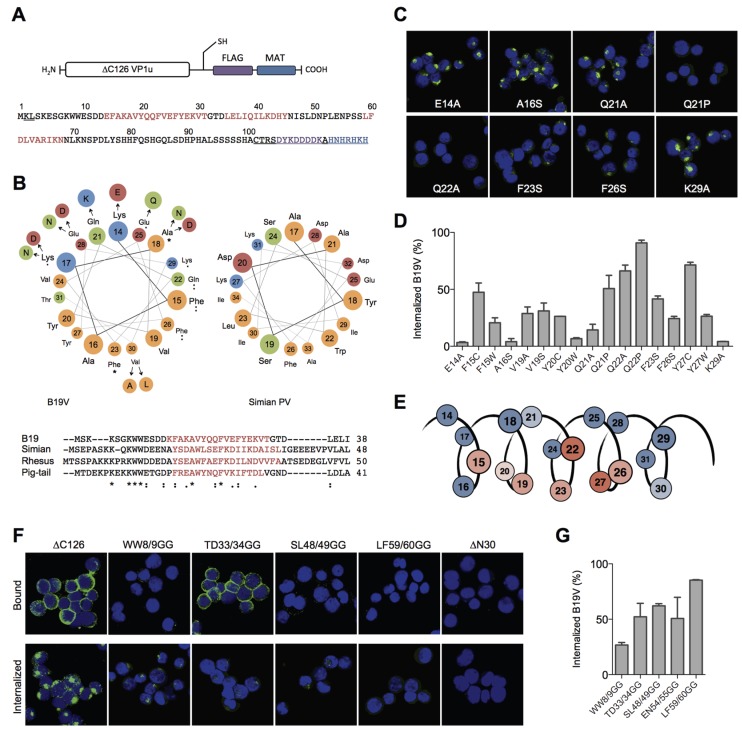
Structural and functional motifs within the RBD. (**A**) Schematic depiction of the recombinant ∆C126 VP1u and its amino acid sequence (AA 1–101 of the VP1u). The sequence of the ∆C126 harbors three predicted helices (red): AA 14–31 (helix 1), 35–45 (helix 2), and 59–68 (helix 3). Sequences of the expression vector (underlined) include the unique cysteine, the FLAG tag (violet), and the metal affinity tag (blue); (**B**) Modeled helical wheel of the conserved helix 1 and the alignment of the erythroparvovirus N-termini. The helical wheel of B19V shows the circular distribution of the AA 14–31 within helix 1 and displays the spatially related clusters of erythroparvovirus-conserved amino acids. Amino acid variations found in B19V isolates are arranged in a wider radius. The amino acids are colored according to their chemical classification: hydrophobic = yellow; polar = green; basic = blue; acid = red. The helical wheel of the simian parvovirus helix 1 is shown on the right side. The erythroparvovirus sequences from B19V, simian, rhesus macaque, and pig-tailed macaque parvovirus were aligned with ClustalW2. The secondary structure prediction with JPred shows the conservation of helix 1 (red) in all four erythroparvovirus members; (**C**) Representative immunofluorescence images of internalized recombinant VP1u mutants (green) into UT7/Epo cells; (**D**) Cellular uptake of B19V in the presence of recombinant VP1u mutants and quantification of internalized B19 virions by qPCR; (**E**) Schematic summary of the obtained data (B–D). The color of amino acids within helix 1 shows their relevance for the VP1u internalization function: red (important) to blue (not relevant); (**F**) Binding and internalization of double-glycine mutants and controls (∆C126, ∆N30); (**G**) B19V internalization in presence of the different GG-mutants. For all qPCR results *n* ≥ 2.

To elucidate structural and functional motifs within the RBD, we performed *in silico* structural predictions and sequential alignments of AA 1–80 of B19V with the VP1u sequences from other erythroparvoviruses (simian, pig-tail and rhesus macaque parvovirus), and from natural B19V isolates. The secondary structure prediction of the RBD with the JPred server [31] indicated three α-helices (red sequence) with high confidence: helix 1 (AA 14–31), helix 2 (35–45), and helix 3 (59–68) (Figure 2A). However, the secondary structure prediction of the other erythroparvoviruses showed only conservation of helix 1 (Figure 2B). Strikingly, the helix 1 of all four erythroparvoviruses revealed a conserved and strong amphiphilic character. 

The sequential alignment of the B19V RBD with VP1u sequences from other erythroparvovirus members showed clusters of conserved amino acids in the very N-terminal part (AA 1–30), including helix 1 (Figure 2B). In contrast, the alignment of the sequence AA 30–80 suggested no significant conservation, showing gapped and fragmented alignment patterns. Considering the erythroparvovirus alignment within helix 1, we found a spatial cluster of conserved amino acids at one amphiphilic interface of the helix: F15, A18, Q22, F23, F26 (Figure 2B). 

The analysis of natural B19V isolates revealed a pattern of frequent mutations in the hydrophilic, but not in the hydrophobic, part of helix 1, including mutation of A18 to hydrophilic amino acids. Taken together, helix 1 shows an overall conserved cluster of the amino acids F15, Q22, F23, and F26. We hypothesized that this conserved cluster has an important role in the VP1u binding and internalization and that those amino acids participate in the interactions of the VP1u with the cellular receptor. To test this hypothesis, we mutated these amino acids and performed binding/internalization assays on UT7/Epo cells. Binding and internalization was detected by immunofluorescence and B19V uptake competition was analyzed by qPCR as described above. The binding, internalization and B19V uptake competition experiments showed correlating and consistent results. A selection of immunofluorescence images from VP1u mutants is shown in Figure 2C; the results from the B19V uptake competition are shown in Figure 2D and Table 1. Figure 2E schematically summarizes the findings, showing the importance of the amino acids within the helix.

The results confirm that the conserved amino acids and an intact hydrophobic part in the helix are necessary for VP1u binding and internalization. The mutations of the conserved amino acids F15C, Q22A, F23S, and F26S, all led to a considerable decrease in VP1u binding/internalization. As expected, mutations in the hydrophilic part of the helix (E14A, Q21A, K29A) had no or only little effect on the VP1u functionality. Surprisingly, the mutations of Y27 had a strong effect on the VP1u function, suggesting an important role of this less-conserved amino acid. Furthermore, the introduction of proline as a helix-breaking amino acid at Q21 (and Q22) led to a dysfunctional VP1u. In contrast, the “structure-neutral” mutation Q21A allowed significant binding/internalization. This finding confirms the predicted existence of a helical structure between AA 14–31, playing a critical role in B19V uptake.

### 3.3. A Fold of Three α-Helices Defines the Structure and Function of the RBD

Besides the conserved helix 1 (AA 14–31), the structural prediction of the RBD indicated two further helices with high confidence (helix 2 = 35–45; helix 3 = 59–68). However, the linear and structural alignment of AA 32–68 with other erythroparvoviruses showed no significant conservation within this sequence. To further elucidate structural features of the RBD, we intended to disturb the tertiary conformation of this domain by site-directed introduction of flexible sequences. To this end, we introduced double-glycine (GG) mutations at the borders or between the three helices, thereby generating flexibility in the tertiary conformation of the helical fold, and tested the internalization capacity of the mutants on UT7/Epo cells. Surprisingly, all GG mutants showed a strongly impaired VP1u internalization and competition with B19V (Figure 2F,G; Table 1) independent from their sequence conservation.

The GG mutants within the central part of the RBD (AA 30–68) had an even stronger effect than the mutation of the conserved double-tryptophan residues at position 8/9, suggesting that flexibility in the helical fold fundamentally impairs the function of the RBD. Interestingly, the GG mutant at position 33/34 showed normal binding at 4 °C but lost this capacity after increasing the temperature to 37 °C, leading to almost no binding and internalization at physiological conditions. Obviously, TD33/34GG is a thermo-sensitive binding mutant whose interaction to the receptor becomes unstable at higher temperature (37 °C). Finally, the mutation of LF59/60 to GG showed the strongest effect of all introduced mutations, suggesting a crucial structural or functional role of one or both of these two amino acids for the RBD internalization function.

**Figure 3:**
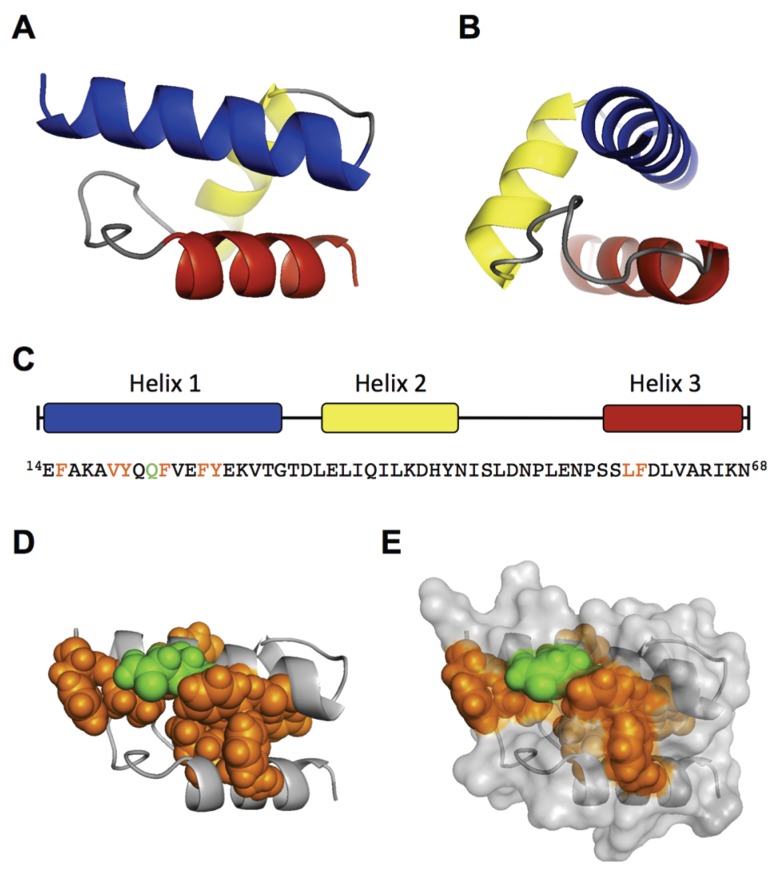
*Ab initio* modeling of the RBD. The central part of the RBD region (AA 14–68) was modeled by QUARK. (**A**) Front view of model 1 with the N-terminus left and C-terminus right. Helix 1 = blue (AA 14–31), helix 2 = yellow (35–45), helix 3 = red (57–68). Helix 1 shows a similar orientation as the schematic depiction in Figure 2E; (**B**) side view of (A), showing helix 1 similar to the helical wheel in Figure 2B; (**C**) Helix distribution and sequence of the modeled AA 14–68. The important amino acids for VP1u internalization are colored in orange (hydrophobic) and green (polar). The spatial cluster of the important amino acids is depicted as spheres in the helical structure (**D**) and in the surface model (**E**) of the RBD. Estimated TM-score of the shown Model 1: 0.5200 ± 0.0833. The structure was visualized with PyMOL.

To support our findings, we performed an *ab initio* modeling of the RBD by the QUARK algorithm [32]. To this end, we first modeled only the sequence from AA 14–68, which includes the three predicted helices. The top 10 output models of the QUARK prediction all showed a similar helical fold of the three helices with a significant confidence (TM-score of Model 1: 0.5200 ± 0.0833) (Figure 3A,B). The main differences between the top 10 models were observed in the coiled structure between helix 2 and 3 (AA 46–56). Modeling of the N-terminal 100 AA (∆C126) led to the same structural core in eight of the top 10 models, differing mainly in the flanking coiled regions (AA 1–13; 69–100). Because the helical fold from AA 14–68 still showed residual internalization capacity in the experiments (Figure 1), the predicted model can be considered as functional structure. Interestingly, the conserved and internalization-relevant amino acids around Q22, Y27 and LF59/60 were modeled in close proximity. This spatial concentration of important amino acids might represent a binding site with the internalization receptor.

Collectively, the experimental results and *in silico* modeling suggest that the RBD has a well-defined structure of a three-helix fold, which forms a spatial cluster of important amino acids at the interface of helix 1 and 3.

### 3.4. Dimerization of the VP1u Enhances the Internalization Efficiency

**Figure 4:**
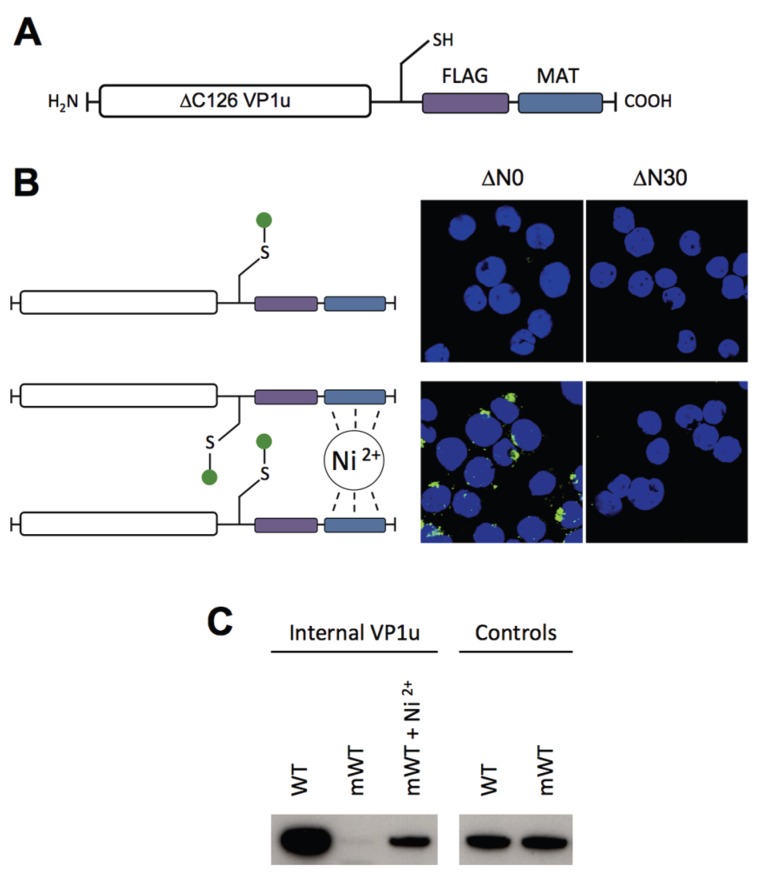
Internalization of VP1u monomer and dimer. (**A**) Recombinant ∆C126 VP1u with unique cysteine (-SH) for specific crosslinking with maleimide-fluorescein; (**B**) Schematic depiction of fluorescein-labeled ∆C126 VP1u (left), which was internalized into UT7/Epo cells and detected by fluorescence microscopy (right). Fluorescein-labeled ∆N30/∆C126 VP1u was used as a negative control; (**C**) Western blot of internalized WT VP1u (WT), monomeric C228G VP1u (mWT), and monomeric VP1u supplemented with 10 µM Ni^2+^. Controls show the relative input of WT and mWT in the internalization experiment.

To have the possibility for specific labeling of the VP1u by a fluorophore, we introduced a unique cysteine in the C-terminal part of the recombinant protein (Figure 4A). However, the labeling of the VP1u by maleimide-fluorescein and internalization of the conjugate on UT7/Epo cells did not result in significant internal fluorescent signal (Figure 4B). Importantly, the chemical modification of the unique cysteine by the maleimide-fluorophore disabled the disulfide bond formation and thus the dimerization of VP1u. Hence, we hypothesized that the dimerization of the VP1u might be important for its internalization capacity. To test this hypothesis, we pre-incubated the fluorophore-labeled VP1u (monomer) with Ni^2+^ ions and assayed for cellular uptake. Ni^2+^ ions are specifically chelated by repetitive histidine residues and thus Ni^2+^ can induce dimerization or trimerization of His-tagged proteins, in this particular case the VP1u [34,35]. Under Ni^2+^-mediated dimerization, we observed a significant internalization of fluorescent signal in UT7/Epo cells, as opposed to samples without Ni^2+^ ions (Figure 4B). Furthermore, we expressed a monomeric mutant without the unique cysteine, tested its internalization capacity and detected internalized VP1u by Western blot. The result shows that the disulfide-forming VP1u (dimer) internalized considerably better into UT7/Epo cells than the monomer (Figure 4C). Strikingly, the addition of Ni^2+^ as a dimerizing agent led to an increased internalization of monomeric VP1u, confirming the previous immunofluorescence result.

**Figure 5:**
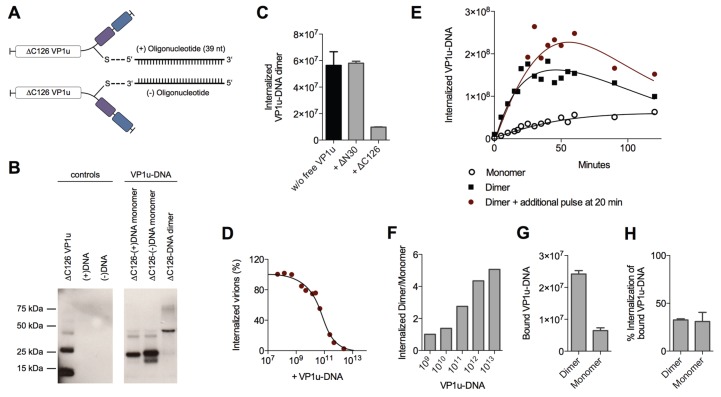
VP1u-DNA as a model for dimerization-enhanced VP1u uptake. (**A**) Schematic depiction of the synthesized ∆C126 VP1u-DNA construct. The unique cysteine of the VP1u was specifically crosslinked with sense(+)- and antisense(−)-oligonucleotides using maleimide and azide/alkyne Click Chemistry; (**B**) Western blot of the ∆C126 VP1u-(+)DNA, the ∆C126 VP1u-(−)DNA, and the hybridized VP1u-DNA dimer (equimolar mixture of ∆C126 VP1u-(+)DNA and ∆C126 VP1u-(−)DNA). Recombinant ∆C126 VP1u was detected by αFLAG antibody. Controls show ∆C126 VP1u without crosslinking and both oligonucleotides (39 nt); (**C**) Internalization of VP1u-DNA dimers into UT7/Epo cells and quantification by qPCR. The internalization (37 °C, 10 min) of 2 × 10^11^ VP1u-DNA dimers was performed in absence (black bar) and in presence of functional (∆C126) or dysfunctional (∆N30) VP1u as competitors; (**D**) Dose-dependent competition of B19V internalization with VP1u-DNA dimer and quantification of internal B19V by qPCR; (**E**) Uptake kinetics of VP1u-DNA monomer (4 × 10^11^) and VP1u-DNA dimer (2 × 10^11^) into UT7/Epo cells. Red graph represents VP1u-DNA dimer internalization where an additional pulse of VP1u-DNA dimer (2 × 10^11^) was added again after 20 min; (**F**) Internalization of different amounts of VP1u-DNA monomer and VP1u-DNA dimer for 5 min. The calculated ratio of detected internal dimer/monomer indicates the dependence of the dimer and monomer detection on the applied VP1u-DNA concentration; (**G**) Binding of VP1u-DNA dimer and monomer on UT7/Epo cells at 4 °C, quantified by qPCR; (**H**) Percentage of the bound VP1u-DNA dimers and monomers shown in (G) that were able to internalize. After the binding and four washes, the samples were incubated at 37 °C for 15 min to allow internalization. Non-internalized particles were removed by trypsinization.

To further study the enhanced internalization by the VP1u dimerization, we sought to develop a sensitive and quantitative assay. To this end, we designed and generated VP1u-DNA conjugates using Click Chemistry, which do not only enable the quantitative detection by qPCR, but also allow the controlled dimerization of the VP1u by DNA hybridization. The unique cysteine of the VP1u was coupled by the heterobifunctional crosslinker maleimide-alkyne to azide-modified sense(+)- or antisense(−)-desoxyoligonucleotides (39 nt) (Figure 5A). The conjugate was purified by Ni-NTA beads and analyzed by SDS PAGE and Western blot (Figure 5B). Dimerization of the VP1u-DNA could be achieved by mixing equimolar amounts of the VP1u-(+)DNA and the VP1u-(−)DNA, thus inducing hybridization of the complementary DNA strands (Figure 5B).

Competition experiments with free VP1u showed that the uptake of the VP1u-DNA dimer was specifically mediated by the VP1u residue (Figure 5C). Furthermore, the VP1u-DNA competed with native B19V internalization into UT7/Epo cells in a dose-dependent manner (Figure 5D). Taken together, these results validate the VP1u-DNA construct as a model for VP1u-mediated uptake of B19V.

To analyze the internalization kinetics of the monomer and dimer VP1u, we incubated UT7/Epo cells with the VP1u-DNA at 37 °C for increasing time periods. After washes and trypsinization, the internalized VP1u-DNA was quantified by qPCR. The obtained kinetics show that the VP1u-DNA dimer internalized considerably faster than the monomeric VP1u (Figure 5E). While the detected signal of internalized VP1u-DNA dimer reaches a plateau after about 30–50 min, the slow uptake of the VP1u-DNA monomer indicates no significant stagnation in the first 120 min. Notably, unprotected DNA is degraded after endocytosis by lysosomal DNases, which can explain the measured decrease of internal VP1u-DNA dimer after the plateau phase. To test whether the stagnation of the measured internal VP1u-DNA dimer is due to a depletion of a cellular factor or the external VP1u-DNA, we added an additional amount of VP1u-DNA after 20 min and measured its internalization (Figure 5E). The results show a further progression of the VP1u-DNA internalization with the additional pulse, suggesting that the cells deplete the extracellular VP1u dimer over time by an efficient uptake.

The depletion of a reaction component and the following reaction stagnation is not only time-dependent but also dependent on the starting quantity of the educt (in this case input of VP1u-DNA). While low input VP1u-DNA quantity leads to fast depletion and stagnation of the uptake reaction, excess VP1u-DNA enable a prolonged and constant internalization reaction. To illustrate this relation between educt input and depletion, we varied the reaction parameter of input VP1u-DNA, while keeping the time of internalization constant at 5 min.

As expected, excess conditions with high input VP1u-DNA (10^13^) indicated a >five-fold difference between internalized VP1u-DNA dimer to monomer (Figure 5F). In contrast, low input concentrations led to a fast stagnation of the efficient VP1u dimer internalization and thus to a similar amount of detected internal VP1u-DNA dimer and monomer.

To elucidate whether the enhanced VP1u dimer internalization is due to an increased binding affinity or due to a more efficient endocytosis, we measured the binding capacity of the VP1u dimer and monomer, and further determined the following internalization of the bound VP1u. The results show that the VP1u-DNA dimer binds significantly better than the monomer (Figure 5G). In contrast to this, the VP1u internalization efficiency of the bound dimer or monomer variants was similar (Figure 5H). In conclusion, the enhanced VP1u dimer internalization compared to the monomer correlates with its increased binding capacity.

## 4. Discussion

The virus internalization into permissive cells is a critical step for the infection. Since this process initiates in the extracellular environment, it is susceptible to interference by the humoral immune response. Insights into the molecular mechanism governing the viral uptake provide not only fundamental information about the viral life cycle, but also significantly contribute to understanding the virus-host immune response and the pathogenesis of the infection. Direct insights into the internalization process of B19V have been limited thus far. The specific study of the internalization step and the involved viral domains would require an approach with mutated recombinant B19V, however, it has been shown that the production of intact virions from a cell culture is considerably impaired [36]. We recently found that the crucial step for B19V internalization into permissive cells is mediated by the immunodominant VP1u region, and that the recombinant VP1u can serve as a model to study viral internalization independently from the complex capsid and the associated difficulties [16,30]. The efficient competition of the recombinant VP1u with the natural B19V uptake validates the VP1u as an appropriate model to study the particular step of viral internalization and further demonstrates that there exists no alternative uptake mechanism for B19V into UT7/Epo cells. Although the VP1u appears to be the crucial region for the internalization of the virus, the influence of the entire B19V capsid should not be underestimated. First, the VP1u-mediated internalization might only represent a single step in a sequence of capsid interactions with cellular receptors, which may also influence the infection and pathogenesis of B19V; second, the capsid structure might affect the conformation and accessibility of the VP1u, thus indirectly modulating the targeting and internalization into susceptible cells [37].

To precisely define the RBD within the VP1u, we introduced serial truncations at the N- and C-terminus of the recombinant protein. These truncations showed that the AA 5–80 are required and sufficient for binding and internalization into susceptible UT7/Epo cells (Figure 1; Table 1). Further truncations from both termini led to a gradual decrease of the binding and internalization capacity, suggesting that the direct interaction to the receptor is mediated by the central sequence of the RBD. The critical region is located between AA 20–68 since further truncations resulted in complete loss of functionality.

To map structural and functional motifs within the RBD of VP1u, we combined sequence and structural alignment of erythroparvoviruses and natural B19V isolates with site-directed mutagenesis (Figure 2). First, we found that the RBD contains a conserved amphiphilic α-helix from AA 14–31 (helix 1), which likely interacts directly with the cellular receptor (Figure 2B). Point mutations within the conserved hydrophobic region of the helix as well as amino acid Q22 resulted in a considerable decrease of the binding and internalization capacity, indicating a possible binding site to the receptor (Figure 2C,D). Second, the results suggest that the non-conserved region from AA 32–68 contains two further α-helices (helix 2 and helix 3), which are assembled with helix 1 in a tightly folded structure. Introduction of flexibility between the helices by double-glycine (GG) mutations fundamentally decreased the VP1u internalization capacity (Figure 2F,G). The introduction of flexibility in the hinge between helix 1 (AA 14–31) and the predicted helix 2 (35–45) resulted in a temperature-sensitive binding mutant. The entropy-dependence of this TD33/34GG mutant suggests that binding to the receptor critically depends on the correct spatial assembly of the three helices as a rigid tertiary structure. Furthermore, the LF59/60GG mutant completely lost the binding and internalization capacity; thus, the amino acids LF59/60 may also be considered as direct binding residues to the receptor. The tertiary structure prediction of the RBD with QUARK reinforced the conclusions from the experimental data, showing a defined tertiary assembly of the three α-helices (Figure 3A). Strikingly, the conserved and functional amino acids of helix 1 were predicted in a spatial cluster with the important amino acids LF59/60 of helix 3 (Figure 3D). It is conceivable that this hydrophobic pocket might represent the binding site to the internalization receptor.

The results show that the hydrophilic and thus immune-accessible region of helix 1 (AA 14–31) is less conserved among B19V isolates (Figure 2B). Mutations in the hydrophilic half of helix 1 are generally well accepted without considerable influence on the VP1u binding and internalization capacity. This finding might represent a possible immune escape mechanism of B19V from neutralizing antibodies against the RBD [38]. Neutralizing antisera against the VP1 revealed a cluster of important epitopes between AA 20–80 of the VP1u [28,29], coinciding with our findings that this region is directly involved in receptor binding (Figure 1). The clustering of strong neutralizing epitopes in the RBD suggests that the interference of the viral uptake plays an important role in B19V neutralization. In line with this notion, the neutralizing antibody N-VP1u from an immune person (Mab-1418-1; epitope AA 30–42) [38,39] inhibited B19V and VP1u internalization into UT7/Epo cells [16,37].

Since all erythroparvoviruses show specificity for the erythroid lineage, it is likely that all members of the genus are using a common VP1u receptor on erythroid progenitor cells. Therefore, the function of the N-terminal VP1u region as RBD should be similar for all erythroparvoviruses. The low sequential and secondary structure conservation of AA 32–68 might suggest that, in fact, this region has the role of stabilizing the correct position and accessibility of the conserved hydrophobic binding pocket at helix 1, rather than being directly involved in the interaction with the receptor. Hence, mutations in this region that significantly disturb the tertiary structure of the RBD are expected to cause loss of VP1u internalization.

Finally, the structural modeling of the RBD might provide important knowledge for the identification of the VP1u receptor, for instance by facilitating the introduction of crosslinking residues at positions close to the receptor binding site.

Using different recombinant VP1u constructs and chemical conjugates, we found that dimerization of the VP1u considerably enhanced the internalization into UT7/Epo cells (Figure 4 and Figure 5). The results show that dimerized VP1u binds more efficiently to the cells compared to the VP1u monomer (Figure 5G), followed by a rapid internalization. Multivalent interaction enhances the binding stability by the increased avidity, which implies that multiple VP1u binding sites can be found in close proximity on the cell surface. Electron microscopy in previous studies has shown that B19V does not require the clustering of multiple viruses to trigger the uptake process [40]. In this context, our findings suggest that multiple VP1 per capsid might initiate a more efficient internalization of B19V into target cells by a multivalent interaction. In fact, B19V capsids contain ~5% VP1 accounting for about three VP1u per capsid [23]. The VP1u regions are likely located close to the fivefold axis of the capsid because the VP2 N-termini of B19 virions were found in the fivefold channel [41]. The cryoelectron microscopy reconstruction has shown that all five VP2 N-termini at one fivefold channel can be exposed at the same time. Therefore, it is possible that all VP1u regions might protrude from the same channel, forming an asymmetrical capsid structure similar to that of many bacteriophages. A localized receptor interaction by an asymmetrical capsid has been proposed for canine parvovirus and might represent a more common phenomenon than previously thought [42]. The simultaneous exposure of the VP1u at the same fivefold channel would induce a dimerization/trimerization similar to the recombinant VP1u-cysteine and the VP1u-DNA conjugate in our study. As opposed to the “asymmetry hypothesis”, it is conceivable that the VP1u has a random distribution in the capsid. Nevertheless, also a distanced distribution of the VP1u might allow multivalent interaction with the VP1u receptors, strengthening the viral binding to the cell by avidity. It is tempting to speculate that the evolution of the virus has favored this multimeric VP1u interaction to the cellular receptors, leading to an enhanced uptake of the virus; however, further studies are required to elucidate this question.

The expression pattern of the receptor globoside [17] or the proposed coreceptors α5β1 [19] and Ku80 [20] does not correlate with the binding pattern of the VP1u [16], suggesting that these receptors are not directly involved in the actual internalization process of B19V. Nevertheless, it is conceivable that the binding of the capsid to additional receptors might stabilize the virus attachment on the cell surface and thus might indirectly facilitate the virus internalization by the VP1u.

## 5. Conclusions

Taken together, we defined the RBD of the VP1u and identified structural and functional motifs within the sequence, which mediate B19V uptake into permissive cells. These findings will contribute to understand the pathogenesis of B19V infection and the molecular mechanisms underlying the clearance of the virus by the immune system.

## Figures and Tables

**Table 1:** Recombinant VP1u mutants generated by site-directed mutagenesis and their remaining internalization capacity on UT7/Epo cells.

Introduced Mutation	B19V Internalization (%) in the Presence of Competing VP1u ^1^	Remaining VP1u Function (%) ^2^
*Truncations*		
WT	4.0	100
∆N5	4.1	99.9
∆N11	17.0	20.5
∆N20	52.9	3.7
∆N30	97.4	0.1
∆C126	3.7	108.4
∆C137	4.5	90.0
∆C147	4.9	81.3
∆C159	28.1	10.8
∆C170	84.8	0.8
		
*Mutations in helix 1*		
E14A	3.4	121.3
F15C	47.5	4.7
F15W	20.6	16.2
A16S	4.1	98.5
V19A	28.8	10.4
V19S	31.1	9.3
Y20C	26.3	11.8
Y20W	6.6	60.1
Q21A	14.4	25.1
Q21P	50.7	4.1
Q22A	66.2	2.2
Q22P	90.8	0.4
F23S	41.6	5.9
F26S	24.4	13.1
Y27C	71.4	1.7
Y27W	26.4	11.8
K29A	4.2	96.4
*Double-glycine mutations*		
WW8/9GG	26.8	11.5
TD33/34GG	52.3	3.9
SL48/49GG	62.1	2.6
EN54/55GG	50.7	4.1
LF59/60GG	85.3	0.7

^1^ Native B19V was internalized into UT7/Epo cells in the presence of a 200-fold excess of VP1u mutants; ^2^ The remaining competitive capacity of the VP1u mutants relative to the WT VP1u was calculated according to the Michaelis-Menten equations for competitive inhibition (competition of native B19V internalization): V = V_max_ × [S] / (K_m_^app^ + 1) with K_m_^app^ = K_m_ × (1 + [I] / K_i_).

## Abbreviations

The following abbreviations are used in this manuscript:

B19V: Parvovirus B19
VP1u: viral protein 1 unique region
RBD: receptor-binding domain
